# Age Matters: Building Blocks Needed to Inform Nurse Staffing Hours Requirements in Residential Care for Older Adults

**DOI:** 10.1093/geroni/igaa057.288

**Published:** 2020-12-16

**Authors:** Heng Wu, Christopher Kelly, Lyn Holley

**Affiliations:** The University of Nebraska at Omaha, Omaha, Nebraska, United States

## Abstract

This study addresses the need for more complete information about the impact of nurse staffing hours (NSH) on nursing home quality of care. We used national data to examine the relationship between three types (Registered Nurse, Licensed Practical Nurse, and Nurse Aide) of hours, and long-stay quality of care measures over time, taking into account the possible confounding influence of regional differences. Data analyzed were from U.S. Nursing Home Compare datasets which reflect quarterly reports, July 1, 2018 - June 30, 2019 (14,768 facilities). The hours for each staff type in each facility were compared with the facility’s four-quarter quality average scores for each of the 12 measures. Results showed only one strong and statistically significant relationship (Beta= .548; p< .001) between Nurse Aide hours and the quality measure used in data sets to exemplify facilities that serve “lower-risk” residents. Analyzes using multiple R (.517) indicate that the linear combination of the three NSH types strongly and significantly (p< .001) predicted the four-quarter average scores and explained 27% of the variance in the scores. Holding the other two NSH types constant, the scores for that measure increased by 63 for each additional increase in the Nurse Aide nurse staffing hours per resident per day. There was no multicollinearity among the three types of staffing hours. This research adds information to the foundation needed for future research about process indicators to assess their efficacy as measures of actual quality of care, and will be submitted as a Technical Note to journals.

